# The combined effect of ultrafine particles of cobalt and manganese oxides and *Origanum vulgare* herb extract on ruminal digestion *in vitro*

**DOI:** 10.14202/vetworld.2024.189-196

**Published:** 2024-01-22

**Authors:** Aina Maratovna Kamirova, Elena Anatolyevna Sizova, Daniil Evgenievich Shoshin, Anastasia Pavlovna Ivanishcheva

**Affiliations:** 1 Federal Research Centre for Biological Systems and Agricultural Technologies of the Russian Academy of Sciences, Orenburg, Russia; 2 Orenburg State University, Orenburg, Russia

**Keywords:** Co_3_O_4_, digestibility, luminescence, Mn_2_O_3_, *Origanum vulgare*, plants, ultrafine particles

## Abstract

**Background and Aim:**

At present, detailed studies are being conducted to confirm the safety of the use of metal-containing ultrafine particles (UFP) in animal feeding, preventing the possibility of negative effects on productive qualities and physiological state, as well as on the environment and final consumer, that is, humans. Thus, the purpose of this research was to study the safety of cobalt- and manganese-containing UFP (UFP Co_3_O_4_, Mn_2_O_3_ UFP) together with *Origanum vulgare* (PB) herb extract in a bioluminescence inhibition test, as well as the effect of this composition on ruminal digestion *in vitro*.

**Materials and Methods:**

The safety of the studied samples was determined using a multifunctional microplate analyzer TECAN Infinite F200 (Tecan Austria GmbH, Austria), recording the luminescence value of the bacterial strain *Escherichia coli K12 TG11* (Ecolum, JSC NVO Immunotech, Russia). Dry matter (DM) digestibility studies were performed using the *in vitro* method on an “artificial rumen” model using an ANKOM Daisy II incubator unit (AD II; USA). The number of protozoa in ruminal fluid was counted in a Goryaev chamber. The bacterial mass was assessed by differential centrifugation followed by drying. This method is based on differences in the sedimentation rate of particles that differ in size and density.

**Results:**

UFP Co_3_O_4_ and Mn_2_O_3_ at concentrations above 1.5 × 10^-5^ and 1.9 × 10^-3^ mol/L, respectively, have a pronounced bactericidal effect, suppressing more than 50% of the luminescence of *E. coli K12 TG1*. The combined use of UFP metals and plant extract increases the luminescence of the test object, indicating its safety. The combined use of UFP and PB increases the digestibility of feed DM *in vitro* and the number of protozoa in 1 mL of ruminal fluid; however, the combination of UFP Mn_2_O_3_ + PB (13.8%) yielded the best result, which is recommended for further *in vivo* research.

**Conclusion:**

*Origanum vulgare* extract reduces the toxicity of UFP Co_3_O_4_ and Mn_2_O_3_ *in vitro*, indicating that their combined use is safer.

## Introduction

In the 21^st^ century, it is a key task of animal science to maximize the genetic potential and preserve the productive longevity of farm animals. In this respect, animal nutrition is important. The efficiency of feed conversion and the profitability of the production of animal products depends not only on the presence and balance of the main nutrients in the diet, such as proteins, fats, carbohydrates, and vitamins but also on the mineral composition. Moreover, not only the actual satisfaction of the need but also the form of the substance is important [1, 2].

Over the past 10 years, many studies have been devoted to the study of mineral nutrition of farm animals and the peculiarities of the formation of the need for elements depending on different factors. A PubMed search query using the keywords “mineral elements, farm animal” shows that the number of related publications exceeds 250,000. The vast majority of these studies have focused on the dose-dependent effects of stimulating metabolic processes and animal productivity [3]. Moreover, parameters such as breed, age, gender, physiological state [4], production technology [5], antagonism between elements [6], sanitary and hygienic conditions of keeping, and environmental quality [7, 8] can significantly influence the need and accumulation of these elements in animal tissues.

Insufficient levels of macro- and micro-elements lead to deterioration of animal health and negatively affect productivity, immunity, and reproduction [9]. At the same time, the chemical structure of the mineral substance used in feeding should be considered [10] to focus on the proven discrepancy in the digestibility of elements by the body from various forms. With high dietary intake and low bioavailability, mineral salts can accumulate in the environment, negatively affect plants and animals [11], affect food chains [12], and ultimately cause symptoms of acute or chronic toxicity [13, 14]. An alternative to mineral salts is the use of ultra-dispersed forms of elements [15].

Thus, the purpose of this research was to study the safety of cobalt- and manganese-containing ultrafine particle (UFP; Co_3_O_4_ and Mn_2_O_3_) together with *Origanum vulgare* (PB) herb extract in a bioluminescence inhibition test, as well as the effect of this composition on ruminal digestion *in vitro*.

## Materials and Methods

### Ethical approval and Informed consent

The study was approved by the Federal Research Center of Biological Systems and Agrotechnologies of the Russian Academy of Sciences, protocol No. 4 dated December 5, 2022. During the studies, measures were taken to ensure a minimum of animal suffering and to reduce the number of experimental samples studied. Verbal informed consent was obtained from all participants before the study.

### Study period and location

The study was conducted from March 2022 to July 2023 in the center “Nanotechnologies in Agriculture” and the “Center for Common Use of the BST RAS”, Orenburg, Russia.

### Experimental design

In the first stage, a series of experiments was carried out to assess the safety of using ultrafine particles (UFP) in the bacterial luminescence inhibition test. In the second stage, the intensity of rumen digestion was determined under *in vitro* conditions.

Chemically pure Mn_2_O_3_ and Co_3_O_4_ UFP (IP Khisamutdinov R.A., Russia) at concentrations of 219.6 and 235.6 mg (laboratory balance VLA, accuracy class I, permissible error ±0.5 mg), respectively, were dispersed in 1 ml of distilled water for 30 min at 25°C. Simultaneously, 20 g of the oregano herb was extracted in a water bath. Subsequently, a series of two-fold dilutions of the obtained components were prepared with dilutions of 2 to 2 × 10^6^.

On the basis of the data of the first stage, the effective concentrations of UFP that suppress 80, 50, and 20% of luminescence as well as those that stimulate it (NTOX+) and the number of dilutions of plant extracts with negative and positive effects were determined. Subsequently, the corresponding suspensions and solutions were combined to determine the mutual effects.

The safety of the test samples was determined on a TECAN Infinite F200 multifunctional microplate analyzer (Tecan Austria GmbH, Austria), recording the luminescence value of the bacterial strain *Escherichia coli K12 TG11* (Ecolum, JSC NVO Immunotech, Russia) in a medium with different contents of UFP and PB for 3 h for 5 min. Distilled water was used as a control substance. On the basis of the data obtained, graphs reflecting the dynamics of bioluminescence inhibition were constructed, and the relative value of bioluminescence was calculated as follows:

A=Io/Ik × 100%,

Where: Ik – Control sample luminosity,

Io – Test sample luminosity.

Dry matter (DM) digestibility studies were performed *in vitro* using an “artificial rumen” model using the ANKOM Daisy II incubator unit (AD II; USA). The incubator has been recognized as an alternative to the traditional *in vitro* procedure. This reduces the need for workforce and increases the number of determinations that a single operator can carry out. The device allows the simultaneous incubation of several feeds in sealed polyester bags in the same incubation vessel, which is constantly rotating at 39.5°C. In this method, the material that disappears from the bag during incubation is considered digested. The method was first developed for the prediction of digestibility of ruminant feeds and has been modified and adapted to improve its accuracy and predictive ability. Modifications used by different researchers include the use of different inocula, buffer solutions, and sample weights.

Rumen fluid was collected through a chronic rumen fistula (ANKOM Technology Corporation, USA) 3 h after feeding the Kazakh white-headed bulls (250 kg, 10 months). The basal diet consisted of 30% concentrates and 70% roughage without UFP and PB addition. Transportation lasted 30 min and maintained a temperature between 38.5 and 39.5°C. The ruminal fluid was stored in a closed container without access to air before analysis. It was shaken thoroughly, filtered through four layers of gauze and incubated in an artificial rumen at a constant temperature of +39.5°C for 48 h.

At the end of the incubation period, the samples were washed and dried at +60°C to a constant weight. The *in vitro* DM digestibility coefficient was calculated using the following formula as the difference in the mass of the food sample with the bag before and after incubation:

K=(A-B)/C × 100%,

Where: K – Coefficient of digestibility of DM of feed, %;

A – Initial weight 1 (feed sample with a bag), mg;
B – Weight after incubation (feed sample with a bag), mg;
C – Initial weight 2 (feed sample without bag mass), mg.

The number of protozoa in the ruminal fluid was determined in a Goryaev chamber. To fix the ciliates, 5 mL of the filtered ruminal contents were removed into a test tube and 0.1 mL of a 4% formaldehyde solution was added. The test tube contents were thoroughly mixed; the liquid was placed in a leukocyte mixer up to mark I and up to mark II – an isotonic sodium chloride solution pre-stained with methylene blue solution. The sample was shaken for 1–2 min, and a 10-fold dilution of the sample was obtained. Ciliates were counted in 100 large squares by introducing one drop of liquid into a chamber with a Goryaev grid under a coverslip. Bacterial weight was assessed by differential centrifugation followed by drying. This method is based on differences in the sedimentation rate of particles with different sizes and densities. The ruminal fluid was centrifuged with a stepwise increase in centrifugal acceleration to deposit a certain fraction at the bottom of the tube at each stage. At the end of each step, the sediment was separated from the supernatant and washed several times to obtain a pure sediment fraction. Centrifuges with a separation factor of approximately 7 thousand (9–10 thousand rpm) were used to sediment the bacteria. Protozoa sedimentation was performed in centrifuges with a low separation factor (1.5–3 thousand rpm). After obtaining a pure sedimentary fraction, it was weighed, and the bacterial weight was determined.

### Statistical analysis

The student’s t-test was used to determine the significance of the differences between the absolute values of bacterial luminescence and DM digestibility coefficients with the required significance level p ≤ 0.01. The tables show the relative values corresponding to this threshold.

## Results

The relative luminescence values of the bacterial strain in a medium with Co_3_O_4_ UFP changed in inverse proportion to its concentration (Table-1). Thus, suspensions containing Co_3_O_4_ UFP 7.8 × 10^-3^; 9.8 × 10^-4^; and 1.5 × 10^-5^ mol/L (hereinafter the molar concentrations are indicated in terms of cobalt) suppressed over 80, 50, and 20% of luminescence, respectively, and 4.8 × 10^-7^ mol/L led to a short-term excess of the control values by 60–90 min of experiment.

However, in the experiment with Mn_2_O_3_ UFP, a lesser bactericidal effect was observed in the absence of absolute inhibition of luminescence. Corresponding effective concentrations: EC_80_ 6.2×10^-2^ mol^/^l, EC_50_ 1.9×10^-3^, EC_20_ 4.9×10^-4^ (Table-2).

**Table-1:** Effect of contact of the bacterial strain *Escherichia coli K12 TG11* with UFP Co_3_O_4_ at various concentrations.

Time (min)	Concentration (mol/L)												

	5 × 10^-1^	6.3 × 10^-2^	3.1 × 10^-2^	7.8 × 10^-3^	1.9 × 10^-3^	9.8 × 10^-4^	4.9 × 10^-4^	1.2 × 10^-4^	6.1 × 10^-5^	1.5 × 10^-5^	7.6 × 10^-6^	9.5 × 10^-7^	4.8 × 10^-7^
0	Tox	Tox	EC_80_	EC_80_	EC_50_	EC_20_	EC_20_	EC_20_	EC_20_	EC_20_	NTOX	NTOX	NTOX
30	Tox	Tox	EC_80_	EC_80_	EC_50_	EC_20_	EC_20_	EC_20_	NTOX	NTOX	NTOX	NTOX	NTOX
60	Tox	Tox	EC_80_	EC_80_	EC_50_	EC_50_	EC_50_	EC_20_	EC_20_	NTOX	NTOX	WNTOX^+^	WNTOX^+^
90	Tox	Tox	EC_80_	EC_80_	C_50_	C_50_	C_50_	EC_20_	EC_20_	EC_20_	NTOX	NTOX	WNTOX^+^
120	Tox	Tox	EC_80_	EC_80_	C_50_	C_50_	C_50_	EC_20_	EC_20_	EC_20_	NTOX	NTOX	NTOX
150	Tox	Tox	EC_80_	EC_80_	C_50_	C_50_	C_50_	EC_20_	EC_20_	EC_20_	NTOX	NTOX	NTOX
180	Tox	Tox	EC_80_	EC_80_	C_50_	C_50_	EC_20_	EC_20_	EC_20_	EC_20_	NTOX	NTOX	NTOX

Numerical values correspond to the value of the relative luminescence value A (%). Color fill – indicators 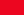 - Tox,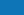 - EC_80_,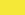 - EC_50_, 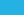 - EC_20_,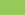 - NTOX, 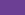
- NTOX^+^that is, the concentration of UFP, causing more than 95, 80, 50 and 20% of biosensor quenching, as well as stimulating luminescence (95 and over 105%) compared to the control

**Table-2:** Effect of contact of the bacterial strain *Escherichia coli K12 TG11* with Mn_2_O_3_ UFP at various concentrations.

Time (min)	Concentration (mol/l)										

	2.5 × 10^-1^	1.2 × 10^-1^	6.2 × 10^-2^	3.1 × 10^-2^	1.6 × 10^-2^	7.8 × 10^-3^	3.9 × 10^-3^	1.9 × 10^-3^	9.8 × 10^-4^	4.9 × 10^-4^	2.4 × 10^-4^
0	C_50_	C_50_	C_50_	C_50_	C_50_	C_50_	C_50_	EC_20_	EC_20_	NTOX	NTOX
30	EC_80_	EC_80_	C_50_	C_50_	C_50_	C_50_	C_50_	EC_20_	EC_20_	EC_20_	NTOX
60	EC_80_	EC_80_	C_50_	C_50_	C_50_	C_50_	C_50_	EC_20_	EC_20_	NTOX	NTOX
90	EC_80_	EC_80_	C_50_	C_50_	C_50_	C_50_	C_50_	EC_20_	EC_20_	NTOX	NTOX
120	EC_80_	EC_80_	C_50_	C_50_	C_50_	C_50_	C_50_	EC_20_	EC_20_	NTOX	NTOX
150	EC_80_	EC_80_	EC_80_	C_50_	C_50_	C_50_	C_50_	C_50_	EC_20_	EC_20_	NTOX
180	EC_80_	EC_80_	EC_80_	EC_80_	C_50_	EC_50_	EC_50_	EC_50_	EC_20_	EC_20_	NTOX

Numerical values correspond to the value of the relative luminescence value A (%). Color fill – indicators 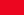 - Tox, 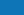 - EC_80_, 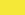 - EC_50_, 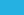 - EC_20_, 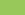 - NTOX that is, the concentration of UFP, causing more than 95, 80, 50 and 20% of biosensor quenching, as well as stimulating luminescence (95 and over 105%) compared to the control

In the case of PB, inhibition of bacterial luminescence was observed only with a 2–8-fold dilution in the first minutes of the experiment, after which the relative value of luminescence in the experimental samples ranged from 150% to 700%, reaching maximum values with a 64-fold dilution of the original extract (Table-3).

**Table-3:** The effect of contact of the bacterial strain *Escherichia coli K12 TG11* with the extract of *Origanum vulgare* herb.

Time (min)	Concentration (mol/L)									

	2	4	8	16	32	64	256	512	2048	4096
0	EC_80_	EC_80_	EC_50_	EC_20_	NTOX	NTOX	WNTOX^+^	WNTOX^+^	WNTOX^+^	NTOX
30	EC_80_	EC_50_	EC_20_	NTOX	WNTOX^+^	WNTOX^+^	NTOX^2+^	NTOX^2+^	WNTOX^+^	NTOX
60	EC_50_	EC_50_	NTOX	WNTOX^+^	NTOX^2+^	NTOX^2+^	NTOX^2+^	NTOX^2+^	WNTOX^+^	NTOX
90	EC_20_	WNTOX^+^	NTOX^2+^	NTOX^2+^	NTOX^2+^	NTOX^3+^	NTOX^3+^	NTOX^2+^	NTOX^2+^	NTOX
120	WNTOX^+^	NTOX^2+^	NTOX^2+^	NTOX^3+^	NTOX^3+^	NTOX^3+^	NTOX^3+^	NTOX^2+^	NTOX^2+^	NTOX
150	NTOX^2+^	NTOX^2+^	NTOX^3+^	NTOX^3+^	NTOX^3+^	NTOX^3+^	NTOX^3+^	NTOX^2+^	WNTOX^+^	NTOX
180	NTOX^3+^	NTOX^3+^	NTOX^3+^	NTOX^3+^	NTOX^3+^	NTOX^3+^	NTOX^3+^	NTOX^2+^	WNTOX^+^	NTOX

Numerical values correspond to the value of the relative luminescence value A (%). Color fill – indicators - EC_80_, - EC_50_, - EC_20_, - NTOX, - NTOX^+^, - NTOX^++^, - NTOX^+++^, that is, the concentration of *Origanum 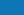
vulgare* 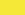
extract, causing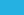 more than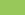 80, 50 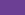
and 20% of 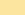
biosensor 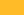
quenching, as well as stimulating luminescence (95, more than 105, more than 150 and more than 300%) compared to the control

**Figure-1:**
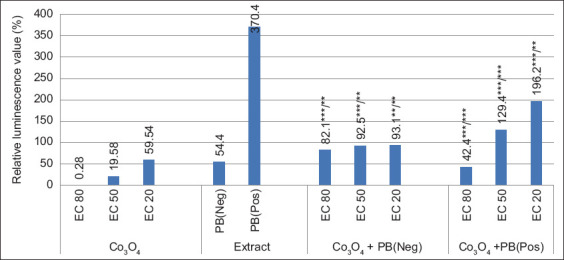
Luminescence dynamics of *Escherichia coli K12 TG1* when Co_3_O_4_ ultrafine particle (UFP) was combined at effective concentrations with PB at inhibitory (Neg) and stimulating (Pos) doses. **p ≤ 0.01; ***p ≤ 0.001 (UFP/PB).

Combining UFP Co_3_O_4_ at concentrations of 7.8 × 10^-3^ (EC_80_); 9.8 × 10^-4^ (EC_50_); 1.5 × 10^-5^ (EC_20_) and 4.8 × 10^-7^ mol^/^l (NTOX+) with PB in 2-fold (Neg) and 64-fold (Pos) dilution, a decrease in the toxicity of UFP Co_3_O_4_ was established (Figure-1). Thus, the previously discovered EC_80_ and EC_50_ indicators during contamination with PB in a 2-fold dilution were observed only for 30–60 min.

**Figure-2:**
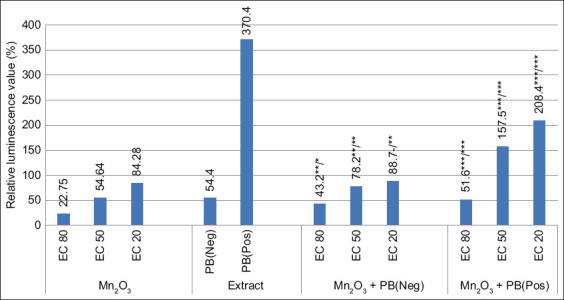
Luminescence dynamics of *Escherichia coli K12 TG1* when combining ultrafine particle (UFP) Mn_2_O_3_ at effective concentrations with PB at inhibitory (Neg) and stimulating (Pos) doses. **p ≤ 0.01; ***p ≤ 0.001 (UFP/PB).

Moreover, the combination of UFP Mn_2_O_3_ with PB (Figure-2) showed the same effect as UFP Co_3_O_4_. However, the absorption capacity of PB (Neg) actually restored the control sample luminescence even at a dose of EC_80_ (82.1%–93.1%). However, in diluted form, PB was more effective against UFP Mn_2_O_3_, but the bactericidal properties remained at the EC_50_ level.

However, the prolonged action of plant extracts for 3 h in all experiments was accompanied by a gradual restoration of luminescence.

### Digestibility of feed DM *in vitro*, population dynamics of protozoa in ruminal fluid and bacterial biomass

UFP Co_3_O_4_ and UFP Mn_2_O_3_ at doses of 1.5 × 10^-5^ and 1.9 × 10^-3^ mol/L increased the digestibility of DM *in vitro* by 5.05 and 4.49%, respectively (p ≤ 0.01). In addition, at the same time, the number of ciliates in 1 ml of ruminal fluid significantly increased – from 233.33 in the control to 344.44 and 555.6 thousand in experimental samples, the bacterial biomass did not undergo significant changes. Smaller doses had a weaker effect, and larger doses slightly reduced the digestibility coefficient (Table-4).

**Table-4:** Digestibility coefficient, number of ciliates and bacterial biomass.

Substance	Concentration (mol/L)	Digestibility coefficient	The number of ciliates in 1 mL of ruminal fluid (thousand pcs)	Bacterial biomass (mg)
Control		63.77 ± 0.92	233.33 ± 11.67	70.8 ± 3.5
UFP Co_3_O_4_	0.8 × 10^-5^	65.21 ± 0.97	244.44 ± 19.56	68.2 ± 5.5
	1.5 × 10^-5^	68.82 ± 0.95**	344.44 ± 24.11*	66.4 ± 4.6
	3.0 × 10^-5^	61.3 ± 1.11	222.22 ± 13.33	69.3 ± 4.2
UFP Mn_2_O_3_	9.8 × 10^-4^	64.12 ± 0.92	233.33 ± 18.67	72.1 ± 5.8
	1.9 × 10^-3^	68.26 ± 0.84**	555.56 ± 38.89*	73.4 ± 5.1
	3.9 × 10^-3^	59.83 ± 0.96**	344.44 ± 20.67	67.1 ± 4

* p ≤ 0.05;

** p ≤ 0.01. UFP=Ultrafine particle

PB increased the digestibility coefficient in all dose variants from 69.36% at 0.5 mL/L to 72.63% at 2.5 mL/L versus 63.77% in the control. The number of ciliates and bacterial mass also increased. In turn, the UFP Mn_2_O_3_ + PB complex increased feed digestibility by 13.78% (p ≤ 0.01), Co_3_O_4_ + PB by 9.07% (Table-5).

**Table-5:** Digestibility coefficient, number of ciliates and bacterial biomass.

Substance	Concentration (mol/L)	Digestibility coefficient	The number of infusoria in 1 mL of scar fluid (thousand pcs)	Bacterial biomass (mg)
Control	-	63.77 ± 0.92	233.33 ± 11.67	70.8 ± 3.5
PB	0.5 mL/L	69.36 ± 1.04**	422.22 ± 33.78**	96.5 ± 7.7*
	2.5 mL/L	72.63 ± 0.99***	555.56 ± 38.89**	87.2 ± 6.1
	5 mL/L	72.53 ± 0.96***	555.56 ± 33.33**	86.3 ± 5.2
UFP Mn_2_O_3_ + PB	9.8 × 10^-4^+ 2.5 mL/L	77.55 ± 1.08***	322.22 ± 22.56*	83.3 ± 5.8
UFP Co_3_O_4_ + PB	1.5 × 10^-5^+ 2.5 mL/L	72.84 ± 0,97***	344.44 ± 20.67*	75.5 ± 4.5

* p ≤ 0.05;

** p ≤ 0.01;

*** p ≤ 0.001. UFP=Ultrafine particle

In summary, free UFP improve feed digestibility *in vitro*. The best results were obtained using a UFP + PB mixture.

## Discussion

The high reactivity of UFP, and therefore its toxicity, is associated primarily with its small size, the ability to penetrate cell membranes and induce reactive oxygen species (ROS) synthesis, which is accompanied by disturbances in DNA repair, transcription, and translation processes, and ultimately leads to cell apoptosis [16], impaired mitochondrial function, and decreased adenosine triphosphate production [17]. In this case, positively charged particles that easily cross cellular barriers and bind to DNA are the most toxic particles [18]. In particular, chernozems contaminated with UFP Co_3_O_4_ are characterized by a decrease in the total number of bacteria, especially the activity of catalase and dehydrogenases [19], and in the freshwater microalga *Chlorella minutissima*, with similar contact, the growth and synthesis of chlorophyll in cells slow down. Moreover, the effective EC_50_ concentrations identified in this case are consistent with those obtained in experiment I and amount to 38.16 ± 1.99 mg/L (≈4.8 × 10^-4^ mol/L) [20].

Similarly, for UFP Mn_2_O_3_, it was found that they reduce oxygen consumption in *Saccharomyces cerevisiae* by 20% at a dose of 50 mg/L and by 50% at a dose of 170 mg/L [21]. Mn_2_O_3_ nanowires have a bactericidal and cytotoxic effect, inhibiting the growth and reproduction of E. coli, as well as the functional activity of mouse C_2_C_12_ myoblasts in an amount of 12.5 μg/ml, which is significantly lower than the established range due to a different physical form [22].

Phytobiotic additives, such as oregano, thyme, cloves, cinnamon, and black pepper, act not only as antibiotic drugs but also as antioxidant components [23], which together improve growth rates, reproductive and immune functions, and reduce methane and ammonia emissions [24] in experiments with food pathogens such as *Staphylococcus aureus*, Listeria spp., *E. coli*, Salmonella spp. [25], Candida [26], and Fusarium [27]. Thus, the use of *Origanum vulgare* essential oil in combination with *Hypericum perforatum*, *Tussilago farfara*, and *Tanacetum vulgare* [28] and in combination with *H. perforatum, T. farfara*, and *T. vulgare* [29] contributed to the survival rate of broiler chickens, increased the average daily growth, and improved the condition of the gastrointestinal tract. At the same time, in calves under the influence of an infusion of oregano flowers, on the 14^th^ day after administration, the concentration of IgG in the blood increased [30], and *in vitro* experiments simulating rumen digestion indicated the formation of more microbial protein and a decrease in methanogenesis [31]. At the same time, oregano is repeatedly mentioned in aquaculture as an effective antioxidant agent that reduces the level of thiobarbituric acids in the blood [32] and improves growth rates, hepatorenal functions, and intestinal morphometry in fish [33].

The high luminescence rate of the recombinant strain *E. coli K12 TG1* can be attributed to the increased sugar and vitamin content in the medium compared with the control. However, further studies of the chemical composition of the plant extract and the dynamics of free radicals in the studied PB-UFP system are required to fully confirm the presented hypothesis.

As mentioned above, the use of essential microelements in UFP feeding has several advantages, in particular, small size and high bioavailability, which make it possible, at minimal cost, to increase the efficiency of growth and physiological functions, reduce feed intake, and improve the quality of agricultural products [34]. For the same reason, oxidative stress may occur accompanied by geno- and cytotoxic effects if the dose is not correctly selected [35].

In particular, the toxicity of UFP Co_3_O_4_, accompanied by an increase in the activity of NADPH oxidase, superoxide dismutase, ROS synthesis, and lipid peroxidation, has been proven in experiments with human lymphocytes and erythrocytes [36], as well as on the example of *Artemia salina* [37] and *Brassica napus* L. [38]. However, both cobalt and manganese play an important role in both the metabolism of animals and plants [39], and their introduction into diets in the form of UFP is justified with the correct dose selection, since they are not only a source of microelements but also an alternative to antibiotics, the problem of resistance that is becoming increasingly relevant [40].

Micronutrient supplementation may negatively affect digestibility and reduce fiber absorption [41]. However, some *in vitro* experiments showed that the addition of Mn_2_O_3_ to rumen microorganism suspension stimulated cellulose digestion, while a high concentration of manganese completely inhibited cellulose digestion. Similarly, Mn_2_O_3_ slightly stimulated the urease activity of rumen microbiota and increased the DM digestibility *in vitro* [42].

Other researchers have observed high fiber digestibility in dairy cows fed mixed chelated minerals (manganese methionine complexes) [43]. Sika deer fed with manganese in the form of manganese methionine exhibit increased digestibility [44]. Some studies have reported significant increases in cellulose intake due to improved growth of rumen cellulolytic micro-organisms or increased metabolic activity [45].

Such results may be dictated, on the one hand, by the increased need of some microbial communities for Co_3_O_4_, and on the other hand, by the formation of cross-links between negatively charged bacteria. Co_3_O_4_ improves body weight gain in cattle when fed hay in combination with urea, which improves cellulose digestion [46]. The daily intake of cobalt chloride (soluble as colactate) can improve the digestibility of rumen fiber [47].

## Conclusion

UFP Co_3_O_4_ and Mn_2_O_3_ at concentrations above 1.5 × 10^-5^ and 1.9 × 10^-3^ mol/L, respectively, have a pronounced bactericidal effect, suppressing more than 50% of the luminescence of *E. coli K12 TG1*. The combined use of UFP metals and PB increases the luminescence of the test object, which indicates the safety of use. The combined use of UFP and PB improved the *in vitro* digestibility of feed DM and the number of protozoa in 1 ml of rumen fluid. In this case, the best effect was achieved when using the UFP Mn_2_O_3_ + PB complex (13.78%), which is recommended for further study *in vivo*.

## Authors’ Contributions

AMK, EAS, DES, and API: Conceptualized and designed the study. AMK, EAS, DES, and API: Prepared the materials and data collection and analysis. AMK: Drafted the manuscript. All the authors have read, reviewed, revised, and approved the final manuscript.
